# Effect of a chemically-modified-curcumin on dental resin biodegradation

**DOI:** 10.3389/froh.2024.1506616

**Published:** 2025-01-14

**Authors:** Qi Dai, Hsi-Ming Lee, Austin Giordano, Fu-Pen Chiang, Stephen G. Walker, Rafael Delgado-Ruiz, Francis Johnson, Lorne M. Golub, Ying Gu

**Affiliations:** ^1^ Department of General Dentistry, School of Dental Medicine, Stony Brook University, Stony Brook, NY, United States; ^2^ Department of Oral Biology and Pathology, School of Dental Medicine, Stony Brook University, Stony Brook, NY, United States; ^3^ Department of Mechanical Engineering, College of Engineering and Applied Sciences, Stony Brook University, Stony Brook, NY, United States; ^4^ Department of Prosthodontics and Digital Technology, School of Dental Medicine, Stony Brook University, Stony Brook, NY, United States; ^5^ Department of Chemistry and Pharmacological Sciences, School of Medicine, Stony Brook University, Stony Brook, NY, United States

**Keywords:** dental caries, dental cavities, dental caries susceptibility, fluoride, *streptococcus mutans*, doxycycline

## Abstract

**Introduction:**

Previous studies have shown *Streptococcus mutans* (*S. mutans*) esterase is a key mediator of dental composite biodegradation, which can contribute to recurrent caries. This study is to investigate the inhibitory effects of a novel Chemically-Modified-Curcumin (CMC 2.24) on esterase activities and related dental material biodegradation.

**Methods:**

Dental adhesive materials and composite resins were incubated in *S. mutans* suspension with CMC 2.24 and other compounds, including doxycycline, Chemically-Modified-Tetracycline (CMT-3), and curcumin for 4 weeks. The pre- and post-incubation surface roughness were evaluated by either laser diffraction pattern and/or a 3D laser scanning microscope. Esterase enzyme inhibition assays were performed with the same test groups and activities were determined spectrophotometrically.

**Results:**

Among all experimental groups, CMC 2.24 significantly reduced surface roughness of dental composite (*p* < 0.01) and adhesive (*p* < 0.01) materials compared to bacteria-only group. Additionally, CMC 2.24 reduced porcine esterase activity by 46.5%, while other compounds showed minimal inhibition. In the *S. mutans* esterase assay, CMC 2.24 showed inhibition of 70.0%, while other compounds showed inhibition ranging from 19% to 36%.

**Conclusion:**

Our study demonstrated that CMC 2.24 inhibited biodegradation of dental composite material more effectively than its mother compound, curcumin. Moreover, the mechanism of this biodegradation was likely mediated through bacterial esterase activity. Doxycycline achieved similar inhibition by completely eradicating *S. mutans* with its antibiotic action; hence, it is not recommended for long-term use.

## Introduction

According to the World Health Organization, dental caries is reported as one of the most prevalent diseases in the world (1). Every year, it is estimated that there are over 100 million patients' dental visits and patient spending over $34 billion to manage recurrent carious lesions (2). Untreated dental caries can be associated with pain and infection which can impact the daily life. The etiology of virgin caries formation is a combination of tooth, cariogenic bacteria, cariogenic diet, and time. The mechanism is the decalcification of the enamel and dentin by pH drop, caused by acidic products from bacterial fermentation of dietary carbohydrates (3). Among the cariogenic bacteria species, *S. mutans* is one of the most prevalent. In the meantime, *S. mutans* has also been shown to be closely related to early childhood caries (ECC) and recurrent caries through its aciduric and acidogenic nature (4). *S. mutans* biodegradation of adhesive or composite resin materials contributes to the restoration failure and the development of recurrent caries (5–7). However, the mechanism(s) of action of the *S. mutans* biodegradation effect discovered previously were still not completely understood (8). Previous research has shown that bacterial esterase played a major role in the degradation process, which contributes to recurrent decay and restoration failure (5, 6, 9–11).

In addition, current non-invasive, non-surgical approaches to managing caries are very limited. Most current therapies are primarily focused on the surgical management of dental caries, which is time consuming and expensive. Topical fluoride and water fluoridation have been proven to be effective in preventing both virgin and recurrent caries (12, 13). However, conservative patients or parents exhibit reluctance towards the application of fluoride (14). New and more efficient treatment options, based on modulation of the *S. mutan*s derived enzyme activities, are in need for the management of dental caries.

Doxycycline, a member in the tetracycline antibiotic class, is widely used as a broad-spectrum bacteriostatic agent (15). Previously, our lab has developed a non-antibiotic dosage, low dose doxycycline as an effective matrix metalloproteinase (MMP) and inflammatory mediator inhibitor (16–18). MMP plays a major role in inflammatory periodontal connective tissue destruction in the human oral cavity and it's one of the main contributors for periodontal disease (19). Chemically-Modified-Tetracycline-3 (CMT-3) was developed as an alternative inhibitor of MMP without the antibiotic ability (20). It is derived from tetracycline hydrochloride and can effectively inhibit MMPs (17, 18). However, concerns regarding antibiotic resistance emerged in the public even when they were administered at non-antibiotic doses (21). Therefore, newer therapeutic agent, Chemically-Modified-Curcumin 2.24 (CMC 2.24), derived from a natural product, curcumin, was developed and it exhibits pleiotropic anti-inflammatory effects as a broad-spectrum MMP modulator, as well as an inhibitor of pro-inflammatory cytokines (22). It acts to resolve inflammation via a multi-target, host-modulatory approach that overcomes the challenges of redundancy, compensation and necessity exhibited by the immune system (23). In pilot studies, it has demonstrated efficacy in a range of animal and tissue culture models of inflammatory diseases. In addition, preliminary safety studies indicate that CMC 2.24 is well-tolerated at concentrations more than 15-times the therapeutic dose when orally administered in a rat model, consistent with the safety profile of its curcumin parent (24).

Thus, the aim of this project is to determine the inhibitory effect of CMC 2.24 on the biodegradation mediated by *Streptococcus mutans*, and the mechanism of action of this bacterial degradation. Eventually, new and more efficient treatment options for management of carious lesions will be developed, and it will change our approach to preventing secondary caries or restoration failures.

## Materials and methods

### Chemical reagents

CMC 2.24 (99.5% pure, MW: 427 g/mol) was synthesized and provided by Chem-Master Intl. Inc. (Stony Brook, NY, USA) and Traverse Biosciences Inc. (Stony Brook, NY, USA). CMT-3 (>96% pure, MW: 371 g/mol) was provided by Collagenex Pharmaceuticals, Inc. (Newtown, PA, USA, now Galderma R&D, Fort Worth, TX, USA), and CMTx Biotech, Inc. (Stony Brook, NY, USA). *S. mutans* strain 700610 (UA159) was purchased from ATCC® (Manassas, VA, USA). All other chemical reagents were purchased from Sigma Chemical Co. (St Louis, MO, USA).

### Composite degradation assay

Dental composite (3M™ Filtek™ Supreme Ultra, St. Paul, MN, USA) disks (8 mm in diameter and 1 mm in height) were made and light-cured against mylar matrix to produce a smooth surface. 100 μl of the stock *S. mutans* was inoculated in 50 ml Brain Heart Infusion broth (BHI, BD Difco™, Franklin Lakes, NJ, USA) media and incubated overnight. The bacteria density of this overnight culture was measured with a spectrophotometer (Bio-Rad SmartSpec 3,000, Hercules, CA, USA) and diluted to 0.08OD with BHI for the incubation. The composite disks were then incubated with the bacteria culture in BHI for 4 weeks in the presence of 5 μM of curcumin, doxycycline, CMT-3, or CMC 2.24, respectively. A negative control group with only sterile BHI media was also included. Groups were in triplicates to eliminate bias (*n* = 3). A prior minimum inhibitory concentration assay was conducted to determine that doxycycline, at 5 μM, would be antibiotic against *S. mutans*. BHI media and experimental compounds were replenished every 48 h. The biofilm on the disks was preserved during aspiration of waste media and no new *S. mutans* was inoculated after the initial inoculation. The biofilm was wiped off prior to the roughness measurement.

Surface roughness of the composite disks before and after the incubation was measured with a 3D scanning laser microscope (Keyence VK-100X, Itasca, IL, USA) and analyzed by VK MultiFileAnalyzer software. Six random spots on each disk surface were chosen to measure the roughness. Standard mean height (sq), the standard deviation of the height distribution of a given surface area, was used as the parameter to quantify the roughness change.

### Adhesive degradation assay

Dental adhesive (3M™ Adper™ Scotchbond, St. Paul, MN, USA) disks (3 mm in diameter and 2 mm in height) were formed in a Teflon ring and light-cured against mylar matrix. Same incubation protocol was used as the composite degradation assay with *S. mutans* and BHI media for 4 weeks. The experimental groups are *S. mutans* only, CMC 2.24, CMT-3, Curcumin, or Doxycycline. All chemical compounds were prepared and used at 5 μM, as with the above experiment. A negative control group with no bacteria was also included.

**Figure 1:**
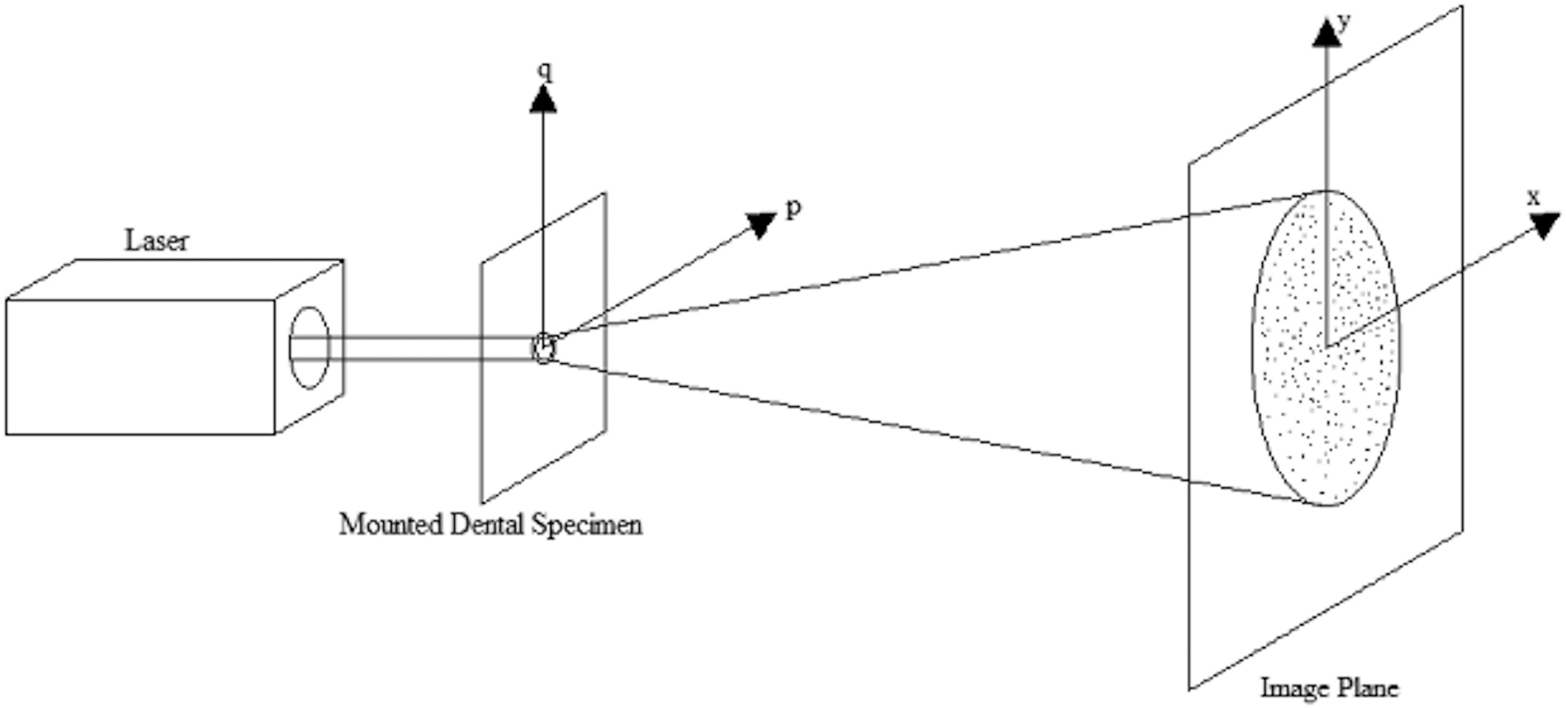
Schematic of the Laser diffractometer.

Besides the measurement method using the 3D scanning laser microscope, laser (ThorLabs HNL020R, Newton, NJ, USA) speckle diffraction pattern analysis was also used to determine the roughness change. Since the adhesive disks are transparent objects, the laser beam could penetrate through them, and a laser speckle pattern generated by the roughness of the adhesive disks could be captured. The readings from before and after the incubation were collected with the laser system and analyzed by the computer software which utilized a Fourier transform to determine the roughness (25). A schematic of the experimental set up and these phenomena are shown in Figure 1. From the laser speckle pattern, the phase, and subsequently the phase difference can be computed at a point *x*. The phase difference is then used to determine the roughness of the adhesive disc as follows:

(1) $$h(x)=\frac{\varphi (x)}{k(n-1)}$$

Where *h*(*x*) is the height of the roughness, *φ*(*x*) is the phase difference of the speckle pattern, *k* is the wave number of the incident light, and *n* is the refractive index of the object. This process is all computed via a MATLAB program developed in-house.

### Esterase activity assay

In order to determine the inhibition of the compounds against pure esterase enzyme, porcine liver esterase (Sigma Aldrich E3019, St. Louis, MO, USA) was used. 1 unit/ml porcine liver esterase, prepared right before the experiment with potassium phosphate buffer, was incubated with the esterase specific substrate p-nitrophenyl butyrate (p-NPB, Sigma Aldrich, St. Louis, MO, USA) for 10 min, with 5 μM of aforementioned chemical compounds as experimental groups (*n* = 3). Enzyme activity was determined by a spectrophotometer (Molecular Devices, SpectraMax® i3x, San Jose, CA, USA) at 405 nm throughout the incubation period.

In order to determine the inhibitory effect of the compounds against *S. mutans* esterase, *S. mutans* suspension was prepared in BHI overnight to reach the desired concentration. It was then spun down at 5,000 rpm. BHI media was aspirated and replaced with potassium phosphate buffer. Subsequently, it was incubated in p-NPB for 12 h with the same experimental groups, in triplicates, as the porcine esterase assay. The same spectrophotometer was used to monitor the activity during the incubation at 405 nm.

### Statistical analysis

For all assays, 2-tailed paired *t*-test was used to determine any statistical significance between the test groups and the control group. The significance level is set to 0.05, meaning any *p*-values less than 0.05 were considered statistically significant.

## Results

**Figure 2:**
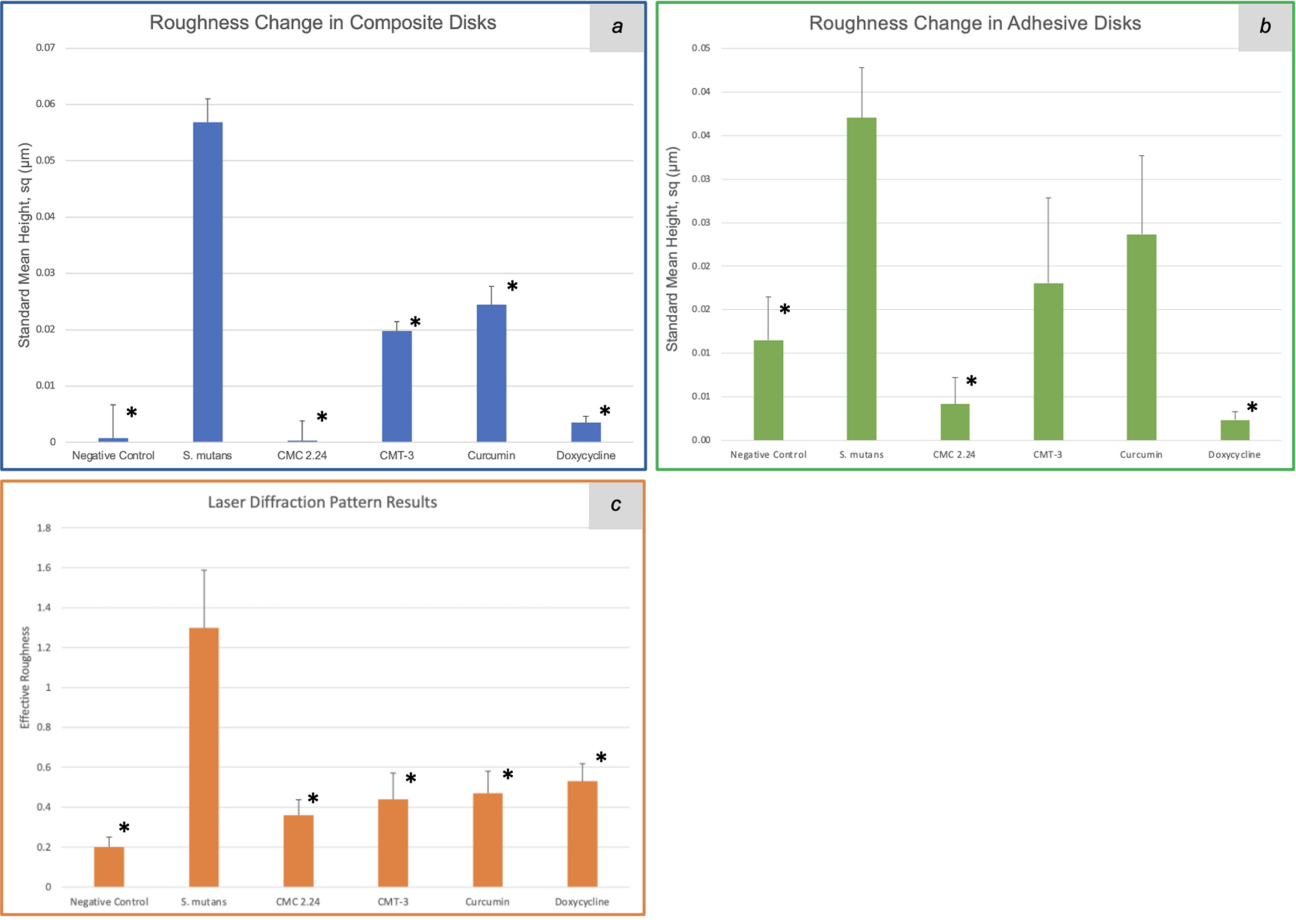
Roughness change obtained with a 3D scanning laser microscope (square mean height) of the composite **(a)** and adhesive **(b)** disk surfaces, and effective roughness obtained with the laser diffraction pattern **(c)** after 4-week incubation with *S. mutans*. *: *p* < 0.05.

**Figure 3:**
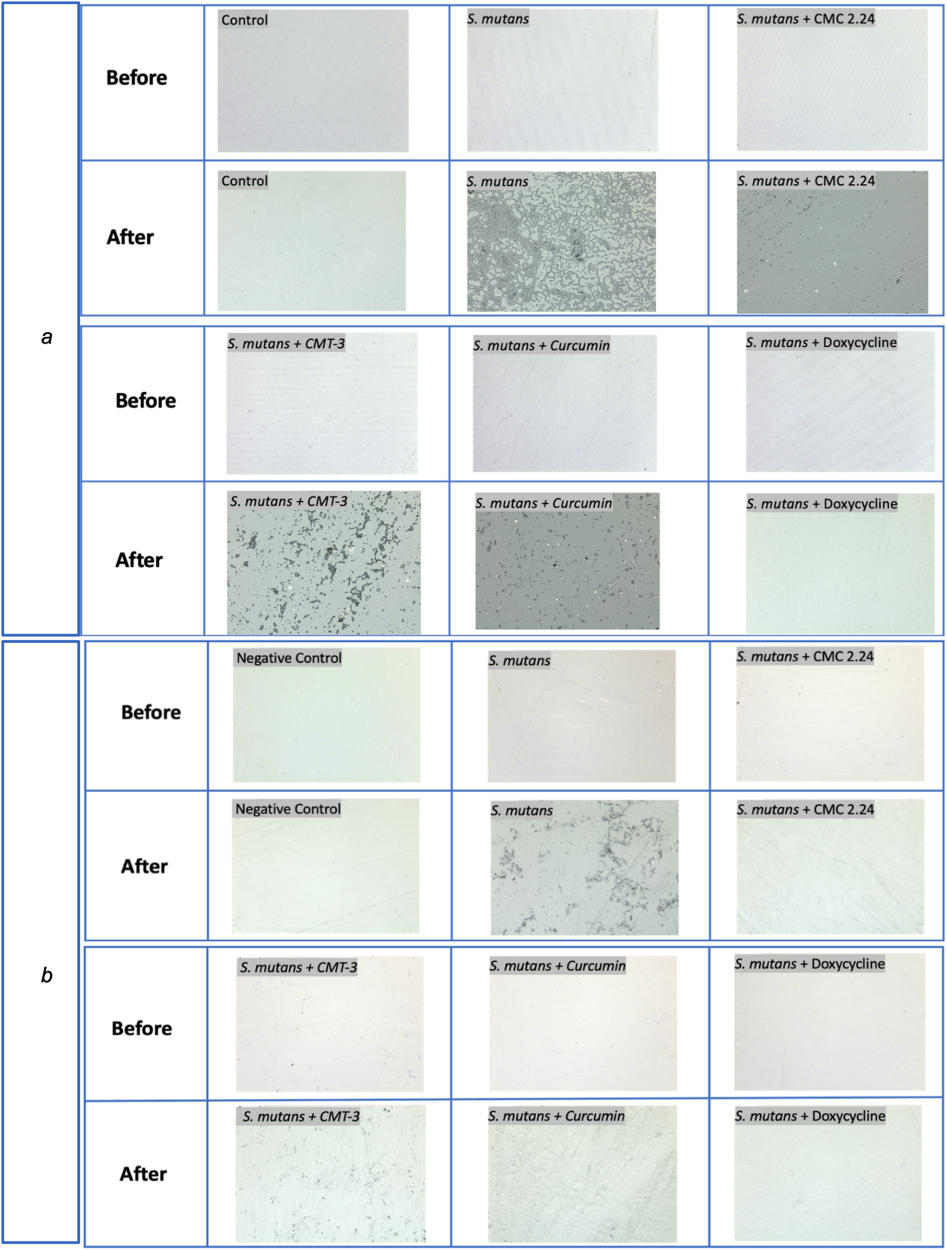
Optical photos captured with Laser microscope for composite **(a)** and adhesive **(b)** disks before and after 4-week incubation with *S. mutans* at 50x magnification.

For the composite degradation assay, the roughness change for the *S. mutans* group was 0.057 μm (standard mean height, sq). The standard mean height encompasses both positive and negative changes observed on a particular surface, reflecting the roughness change in both directions. The roughness change (sq) for the negative control, CMC 2.24, CMT-3, curcumin, and doxycycline were 0.001 μm (*p* = 0.03), 0.0003 μm (*p* = 0.006), 0.02 μm (*p* = 0.008), 0.024 μm (*p* = 0.01), and 0.004 μm (*p* = 0.03), respectively. The results were also summarized in a bar graph in Figure 2a. All of the four test groups were considered statistically significant when compared to the *S. mutans* group (*p* < 0.05). The microscopic photos (50x magnification) of the disk surfaces are shown in Figure 3a. Throughout the incubation period, there was no disturbance in bacterial biofilm formation for all groups except the doxycycline group. Since 5 μM doxycycline is at the antimicrobial level, *S. mutans* growth was inhibited starting from the second day of incubation until the 28th day.

Similar observations were found during the incubation of adhesive disks that there was no disturbance in biofilm formation for all groups except the doxycycline group. The roughness change (sq) measured with the 3D laser microscope for the negative control, bacteria only, CMC 2.24, CMT-3, curcumin, and doxycycline were 0.012 μm (*p* = 0.015), 0.037 μm, 0.003 μm (*p* = 0.002), 0.01 μm (*p* = 0.15), 0.009 μm (*p* = 0.26), and 0.001 μm (*p* = 0.001), respectively, when compared to the *S. mutans* group. The results from the CMT-3 and Curcumin groups were not statistically significant. The roughness change measured with the laser diffraction pattern for the negative control, bacteria only, CMC 2.24, CMT-3, curcumin, and doxycycline were 0.2 (*p* = 0.030), 1.3, 0.36 (*p* = 0.042), 0.44 (*p* = 0.035), 0.47 (*p* = 0.035), and 0.53 (*p* = 0.042), respectively. All groups were statistically significant when compared to the bacteria only group, with a confidence level set to 95%. The results from the laser scanning microscope and the laser diffraction pattern were summarized in Figures 2b,c. The optical images were shown in Figure 3b.

**Figure 4:**
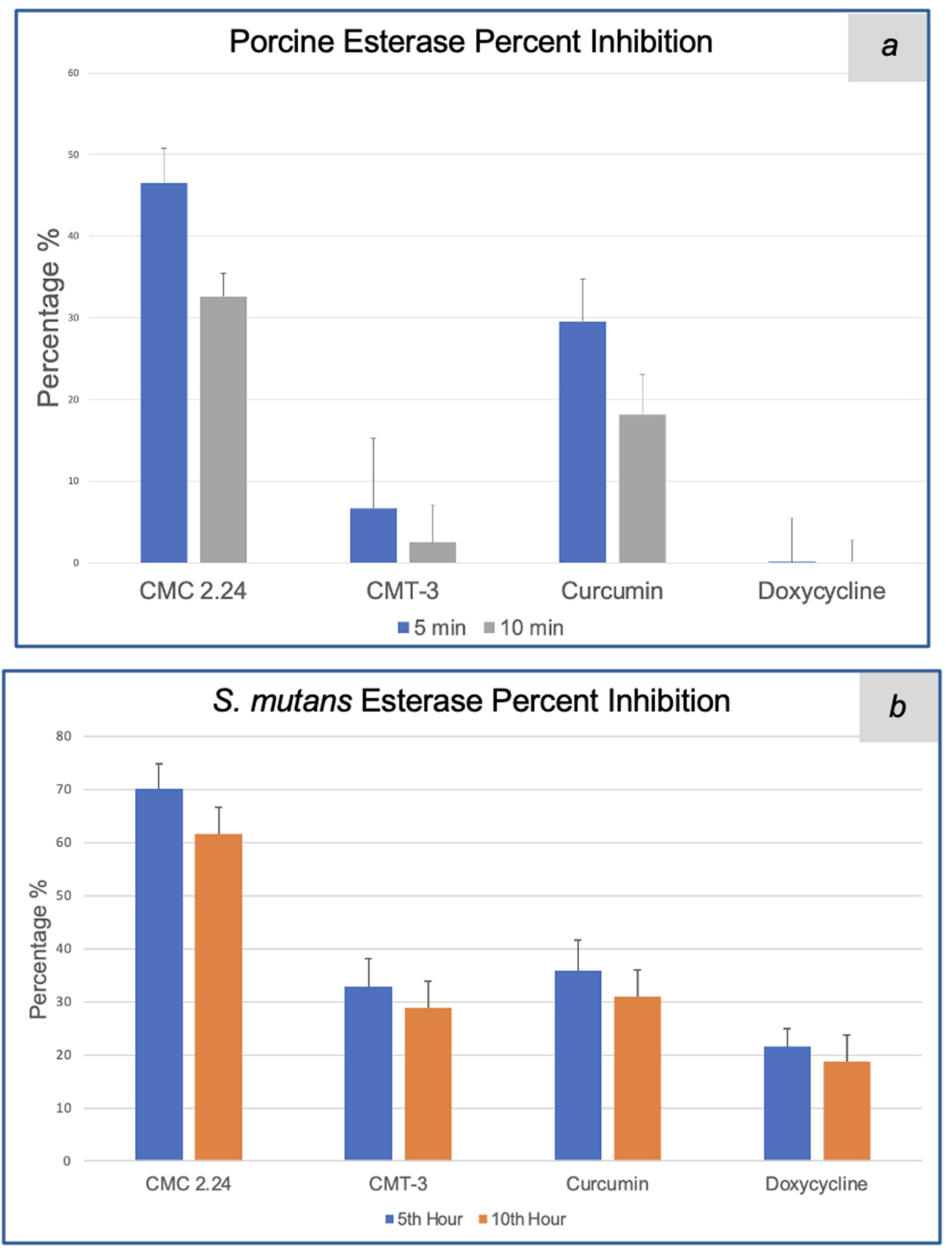
**(a)** Percent inhibition of CMC 2.24, CMT-3, curcumin and doxycycline against pure porcine esterase activity at 5th and 10th min after incubation. **(b)** Percent inhibition of CMC 2.24, CMT-3, curcumin and doxycycline against *S. mutans* esterase activity at 5th and 10th hour after incubation.

For the porcine esterase assay, enzyme activities with various inhibitors were recorded every minute. The results from the 5th and 10th min were analyzed for percent inhibition. At the 5th min, CMC 2.24 had a percent inhibition of 47% against pure porcine esterase, while CMT-3, curcumin, and doxycycline had 7%, 30% and 0% inhibition, respectively. At the 10th min, CMC 2.24 had a percent inhibition of 33% against pure porcine esterase, while CMT-3, curcumin, and doxycycline had 2%, 18% and 0% inhibition, respectively. The bar graph summarizing the percent inhibition is shown in Figure 4a.

In terms of the *S. mutans* esterase assay, enzyme activities with various inhibitors were recorded every 30 min. The results from the 5th and 10th hour were analyzed for percent inhibition. At the 5th hour, CMC 2.24 had a percent inhibition of 70% against *S. mutans* esterase, while CMT-3, curcumin, and doxycycline had 33%, 36%, and 22% inhibition, respectively. At the 10th min, CMC 2.24 had a percent inhibition of 62% against pure porcine esterase, while CMT-3, curcumin, and doxycycline had 29%, 31%, and 19% inhibition, respectively. The bar graph summarizing the percent inhibition is shown in Figure 4b.

## Discussion

Previous studies demonstrated that the biodegradation of dental adhesive and composite materials were mediated by *S. mutans* esterase activities (5, 6, 9). In this study, all three compounds (CMC 2.24, CMT-3, and curcumin) exhibited inhibition against *S. mutans* esterase mediated biodegradation. CMC 2.24 performed the best among the test groups by significantly reducing roughness change on composite and adhesive disks. It demonstrated the greatest inhibition against the esterase degradation of composite and adhesive dental materials, better than its mother compound curcumin. Similar results were found in the doxycycline group. However, doxycycline achieved its inhibition through its antibiotic effect by eliminating *S. mutans* entirely, serving as the positive control for the study. The capability of *S. mutans* to directly break down composite material has played a major role in the development of recurrent caries (26). However, long term use of antibiotic medications can lead to adverse effects, including an increased risk of antibiotic resistance, among others, making it unwise to employ antibiotics for the sole purpose of preventing caries (27).

In evaluating the degradation and surface roughness of adhesive materials, two detection methods were employed: 3D scanning laser microscopic analysis and laser speckle diffraction pattern analysis. No previous studies have used both methods together to analyze the roughness of dental materials. This study provides new insights into the evaluation and measurement of dental material roughness by combining the two techniques. The 3D scanning laser microscope is primarily used to evaluate surfaces, whether transparent or opaque, while the laser speckle diffraction pattern analysis is limited to transparent objects. Therefore, using data from one method to verify the results from the other offers a significant advantage. Minor discrepancies were observed between the results from the two methods, likely due to variations in the collection points across the disk surface. In other words, the slight differences in outcomes can be attributed to the use of distinct reading areas with different equipment. Nonetheless, the laser diffraction pattern analysis provided an additional means of observing and measuring changes in roughness.

For the second part of this study, CMC 2.24 also showed the greatest inhibition against porcine esterase and *S. mutans* esterase activity. This in turn, confirmed that the inhibition of composite degradation by CMC 2.24, as identified in the initial phase, was through inhibiting *S. mutans* esterase activity. Interestingly, doxycycline did not inhibit porcine esterase activity at all, and the minimal inhibition against *S. mutans* esterase was mostly due to its antibiotic nature. If this esterase inhibition is the major inhibitory contributor toward the decreased biodegradation of dental composite material, it would represent a novel approach to preventing the formation of recurrent caries.

Currently the most common method of caries prevention on the market is fluoride treatment. Both topical fluoride and water fluoridation at appropriate concentrations have been proven safe and effective (28). However, concerns about fluoride toxicity that could cause organ damage or even affect cognition and intelligence have arisen (29). This has led to more hesitancy in using fluoride and lead to patients having more caries clinically, leading to increased time and financial costs for treatments. In order to encourage more patients to use fluoride to prevent caries, education is key, especially for parents. However, even with education, many patients are still concerned and unwilling to follow clinicians' recommendations. Therefore, alternatives to fluoride which could provide the same protective effects but without the fear of toxicity are urgently needed to mitigate caries prevalence. Currently, a nanohydroxyapatite toothpaste is on the market as a replacement for fluoride toothpaste, but toxicity concerns are not completely eliminated (30, 31). Further research studies are needed to validate an appropriate concentration that ensures both efficacy and safety for its reliable use. The findings of this study, which demonstrate the caries prevention capabilities of CMC 2.24, have uncovered yet another potential application for this compound.

In addition, there is limited research investigating the etiology of recurrent or secondary caries (32). When assessing the impact of various restorative materials on the occurrence of recurrent caries, a conclusion cannot be drawn to say one material is more superior than the other, when comparing glass ionomer, composite, or amalgam restorations (33). Preventing recurrent caries relies on employing the same methods used to address virgin caries, as there aren't distinct mechanisms dedicated solely to this purpose. Even restorative materials designed to release fluoride have proven ineffective in preventing secondary decay (34). The primary means of managing recurrent decay is still through the mechanical removal of failed restorations and caries, with less emphasis placed on preventative measures (35). By inhibiting esterase activity, CMC 2.24 has the potential to reduce the degradation of composite material at the interface between the tooth and the restoration, thereby preventing the separation between the two. This novel mechanism proposed could transform the way clinicians manage secondary decay.

In our previous studies, CMC 2.24 has demonstrated the safety and efficacy in numerous animal studies. Future clinical trials will be needed to evaluate its safety and efficacy in human (36, 37). However, as it's derived from a natural product like curcumin, it can potentially be easier for patients to accept. One common aspect of these studies was that the systemic administration of CMC 2.24 through oral ingestion (38). This study on caries prevention has opened another approach for the application of this novel CMC. Topical application with toothpaste or mouth rinse containing CMC 2.24 can be an efficient delivery mechanism to prevent recurrent caries.

## Conclusion

This study demonstrated that CMC 2.24 more effectively inhibited biodegradation of dental composite material than its mother compound, curcumin. Moreover, the mechanism of this inhibition of biodegradation was likely through inhibiting bacterial esterase activity. This novel CMC exhibited significant therapeutic potential for decreasing recurrent caries by stabilizing the tooth-restoration interface from *S. mutans* esterase activity.

## Data Availability

The raw data supporting the conclusions of this article will be made available by the authors, without undue reservation.
